# Comparison of Clinical Symptoms and Neurophysiological Findings in Patients With Chemotherapy Induced Peripheral Neuropathy

**DOI:** 10.3389/fneur.2022.838302

**Published:** 2022-06-01

**Authors:** Kye Hee Cho, Eun Young Han, Ji Cheol Shin, Min Cheol Ha, Kwang Ho Ahn, Su Hyun Cho, Sang Hee IM

**Affiliations:** ^1^Department of Rehabilitation Medicine, CHA Ilsan Medical Center, CHA University School of Medicine, Goyang, South Korea; ^2^Department of Rehabilitation Medicine, Jeju National University School of Medicine, Jeju, South Korea; ^3^Department and Research Institute of Rehabilitation Medicine, Severance Hospital, Yonsei University College of Medicine, Seoul, South Korea

**Keywords:** chemotherapy-induced peripheral neuropathy, nerve conduction study, sensitivity, cancer, taxane

## Abstract

**Introduction:**

Taxanes are associated with a distal sensory neuropathy, significantly affecting cancer survivor quality of life. However, chemotherapy-induced peripheral neuropathy (CIPN) assessments are primarily based on clinical symptoms rather than objective neurophysiologic findings. Therefore, we investigated neurophysiologic changes in symptomatic subjects, comparing them with symptom severity.

**Materials and Methods:**

Medical charts of 111 subjects who were referred for CIPN diagnosis after chemotherapy for breast or ovarian cancer between May 1, 2016, and December 31, 2019, were retrospectively reviewed. Demographics, anthropometric parameters, and Leeds Assessment of Neuropathic Symptoms and Signs (LANSS) pain scale data were collected. The nerve conduction study (NCS) results, including sensory nerve action potentials recorded from sural nerves, were analyzed relative to clinical symptoms. To optimize follow-up (FU) NCS diagnostic sensitivity, relative references of FU sural amplitude reductions to >30% and an absolute reference <10 μV were used.

**Results:**

Eighty-eight female patients met the criteria, and 20 underwent FU NCS. Baseline and FU sural amplitudes showed significant positive correlation with respective LANSS scores (*p* < 0.01). FU sural amplitude was significantly lower than the initial result (*p* < 0.05). The FU LANSS score was not different from the initial score. Initial NCS sensitivity and specificity for clinically suspected CIPN diagnoses with LANSS were 69.7 and 47.3%, respectively. All 20 subjects with FU evaluation were clinically compatible with CIPN (LANSS >12) at initial and FU assessments. Among them, only six (30.0%) had abnormal sural amplitudes (<10μV for ≤50 s, <3 μV for 60 s, <1 μV for 70 s) in the initial NCS. In the FU NCS, sural amplitude became abnormal in five additional subjects. Between the initial and FU NCS, sural amplitude was reduced by > 30% in eight subjects (40.0%). NCS sensitivity increased to 65.0% when including either abnormal sural amplitudes or a > 30% reduction in sural amplitude in FU studies.

**Conclusions:**

Although clinical symptoms and NCS results were positively correlated, a single NCS point had limited value for suspected CIPN electrophysiological diagnoses. Serial NCS during chemotherapy might help assess the degree of chemotherapy-induced nerve damage, attain evidence of CIPN prior to symptom aggravation, and monitor the progression of CIPN. Further study is needed to find specific relative references for variable patient factors to increase the sensitivity of electrophysiological studies of clinically suspected CIPN.

## Introduction

Chemotherapy agents prevent cancer progression by inhibiting rapid cancer cell division (1). However, several of these agents are neurotoxic and cause motor, sensory, and autonomic nerve damage (2). Side effects associated with nerve damage include myalgia, fatigue, gait difficulty, peripheral edema, secondary injury from hypoesthesia and allodynia, sleep deprivation, and loss of independence in activities of daily living, which often cause unsolicited discontinuation of chemotherapy or dosage restriction (3). Taxanes are known for such neurotoxicity by damaging Schwann cells and axons in in-vivo & in-vitro experiments, however, the exact mechanism has not been clearly elucidated (4).

Chemotherapy induced peripheral neuropathy (CIPN) is mostly related with an axonal degeneration and the “dying back” axon degeneration of distal nerve endings (5, 6). Although some of the neuropathy processes induced by chemotherapy is reversible, in up to 40% of cancer survivors, the damage is irreversible which makes it crucial to diagnose and control CIPN in the early stage (7). However, precise methods for early diagnosis of CIPN are not established, and the diagnosis is mostly based on clinical symptoms. Although the electrophysiological test involving nerve conduction study (NCS) is a standard diagnostic method, it is often insensitive in detecting early stage CIPN, even when sensory or motor symptoms are present (8). Only few researchers have attempted to explain the relationship between the clinical features and neurophysiologic changes of CIPN (9).

Therefore, this retrospective study aimed to compare symptom severity and neurophysiologic findings and to confirm the feasibility of NCS for diagnosis and monitoring of CIPN in symptomatic subjects during or after the chemotherapy.

## Materials and Methods

### Study Population

The medical charts of 111 subjects with breast or ovarian cancers who visited a tertiary University hospital for sensory discomfort after chemotherapy between May 1, 2016, and December 31, 2019, were reviewed.

Inclusion criteria were (1) history of completion of at least four cycles of chemotherapy with taxanes under the diagnosis of breast cancer or ovarian cancer, (2) complaints of sensory symptoms in glove and stocking distribution compatible with neuropathic pain lasting for more than one week, (3) those who had undergone NCS during or after the chemotherapy. Subjects were excluded if they had (1) sensory symptom before chemotherapy, (2) predisposing conditions for neuropathy, such as diabetes mellitus, thyroid disease, alcohol abuse history, long-term steroid use of more than 3 months, (3) history of focal neuropathy such as carpal tunnel syndrome, cubital tunnel syndrome, and (4) previous chemotherapy for other malignancies. Demographics and clinical features were collected along with parameters of body mass index, the regimen, and the number of chemotherapies, Leeds Assessment of Neuropathic Symptoms and Signs (LANSS) pain scale, and the sensory nerve action potential (SNAP) recorded in the sural nerve.

Finally, 88 patients were included in this study and 23 patients were excluded: 13 with history of diabetes mellitus, thyroid disease, or long-term use of steroid more the 3 months, 5 patients with previous carpal tunnel syndrome and 5 patients with previous chemotherapy with other malignancy or without taxanes. Because all of the patients had been referred for a CIPN diagnosis, their first LANSS and NCS were assessed before any medications were provided. Individuals with deteriorating symptoms during follow-up (FU) were assessed for the FU LANSS and NCS, regardless of oral medication or physical modalities for neuropathic symptoms.

### Clinical Evaluation

Clinical symptom was evaluated using the LANSS pain scale (10). The LANSS is a screening tool for identifying symptoms and signs of neuropathy, composed of sensory description analysis and sensory dysfunction examination (11). It consists of 7-item pain scales grouped into two parts. The first part is five items of self-reported pain description, and the second part is two items assessing sensory dysfunction for allodynia and altered pinprick threshold. The maximum score of 24 points composes of 16 points in the sensory description pain questionnaire and 8 points in the sensory dysfunction examination. LANSS score over 12 was considered clinically compatible with CIPN (10, 12). The purpose of this scale is to assess whether neuropathic mechanisms contribute to the patients' pain. A total score of <12 indicates that the neuropathic mechanism is unlikely to contribute to the pain, and scores more than or equal to 12 indicate that the neuropathic mechanism is likely to contribute to the pain.

### Nerve Conduction Study

Neurophysiological evaluation included measurement of the sensory NCS in median, ulnar and sural nerves at bilateral upper and lower extremities. The sensory nerve conduction studies including median, ulnar, and sural nerves were recorded in orthodromic manner. For sural nerve conduction, patients were asked to lie on stomach while their feet hang over the edge of the bed. The active electrode was placed posteroinferior to the lateral malleolus, and the reference electrode was placed at least 4 cm distal to the active electrode. The ground electrode was placed between the active electrode and stimulation site. The stimulus was given at 14 cm proximal to the active electrode, slightly lateral to the midline of the posterior calf. The responses were averaged 3 times to minimize the effect of the background noise on the waveforms. The skin temperature was monitored during the study to be between 32°C−37°C. As CIPN involves primarily length-dependent axonal degeneration (5), the amplitude of the sural NCS, the longest sensory nerve in human, was used to assess CIPN in this study (13, 14).

There are two main methods of interpreting NCS results: one is applying absolute reference values and the other is relative comparison of the values of the affected and the non-affected sides (15). Since CIPN involves bilateral limbs, relative comparison of each side can be unreliable. Therefore, absolute reference values were applied for diagnosis of CIPN. As the standard sural amplitude is 10–50 μV (8), sural amplitude <10 μV was used as a diagnostic criterion. Moreover, in consideration of age difference in sural amplitude, 3 μV and 1 μV was used as the lower limits for 60 s and 70 s, respectively based on a study of healthy elderly (16) (Criterion A: <10 μV for ≤50 s, <3 μV for 60 s, <1 μV for 70 s). In addition, a decrease in sural amplitude from the initial value at FU was included as another diagnostic criterion (Criterion B) based on a previous study which identified > 30% drop in sural amplitude as an independent risk factor for developing CIPN in colorectal cancer patients (17).

### Statistical Analysis

Results of NCS were analyzed in relation to the clinical symptoms. The data were analyzed using 2 × 2 tables to determine the sensitivity and specificity of the NCS for the diagnosis of CIPN depending on LANSS score. Sensitivity was calculated as the proportion of true positive CIPN on NCS in the patients with LANSS of 12 or more. Specificity was calculated as the proportion of true negative tests on NCS in patients with LANSS <12. In addition, for 20 patients with FU NCS, NCS changes were analyzed with clinical symptoms on FU LANSS. Pearson correlation analysis was performed for correlation between NCS results and LANSS scores. The statistical analysis was performed using standard statistical software (SPSS version 21.1 for Windows, SPSS, Inc., Chicago, IL, USA). The significance level was set at 0.05.

## Result

A total of 88 patients (mean age 51.7 ± 11.3 years) were eligible for analysis. Demographics and baseline characteristics are described in Table 1. The first NCS and LANSS scores were assessed after (mean duration 128 ± 56.6 days) the initiation of chemotherapy, while FU NCS evaluations were available in 20 patients. The mean duration of FU NCS was 62.7 ± 54.2 days 2 months after the initial assessment (Table 1).

**Table 1:** Demographic and clinical characteristics (*n* = 88).

**Characteristics**	***n* (%)**
Age (years)	51.7 ± 11.3
Weight (kg)	61.2 ± 10.1
BMI (kg/m^2^)	24.3 ± 3.5
Cause, *n* (%)	
Ovarian cancer	30 (34.0)
Breast cancer	58 (66.0)
Additional CTx regimen to Taxanes	
Cyclophosphamide	6 (6.8)
Platin	31 (35.2)
Adriamycin + Cyclophosphamide	51 (58.0)
Duration between 1st CTx and NCS (days)	128 ± 56.6
Duration between 1st and 2nd NCS (days)	62.7 ± 54.2
Amplitude of sural SNAP (μV)	
Initial (*N =* 88)	11.8 ± 8.1
FU group (*N =* 20)	
Initial	11.4 ± 7.0
FU	8.8 ± 6.1^**^
LANSS	
Initial (*N =* 88)	11.4 ± 5.2
FU group (*N =* 20)	
Initial	16.0 ± 2.0
FU	16.4 ± 2.0

*Values are mean ± standard deviation or numbers (%)*.

** *p value < 0.01, compared to initial amplitude*.

*BMI, body mass index; CTx, chemotherapy; SNAP, sensory nerve action potential; NCS, nerve conduction study; FU, Follow-up; LANSS, Leeds Assessment of Neuropathic Symptoms and Signs*.

The average sural SNAP amplitude of 88 patients was 11.8 ± 8.1 μV. In 20 patients with FU NCS studies, the mean values of the initial and FU sural amplitudes were 11.4 ± 7.0 and 8.8 ± 6.1 μV, respectively. The initial and FU amplitudes of sural SNAP showed a significant correlation (*p* < 0.01). The sural amplitude was significantly reduced at FU NCS by 22.8% (*p* < 0.01) (Table 1). The average LANSS score for 88 patients was 11.4 ± 5.2. Initially, 42 of 88 patients were clinically compatible with CIPN based on a LANSS score over 12. Only 23 among 42 patients with abnormal LANSS had abnormal sural SNAP amplitude (Criterion A) in the NCS. Among 36 patients with normal LANSS, 26 patients showed negative results in the NCS. In other words, the sensitivity and specificity of the initial NCS for clinically suspected CIPN were 69.7 and 47.2%, respectively.

**Figure 1:**
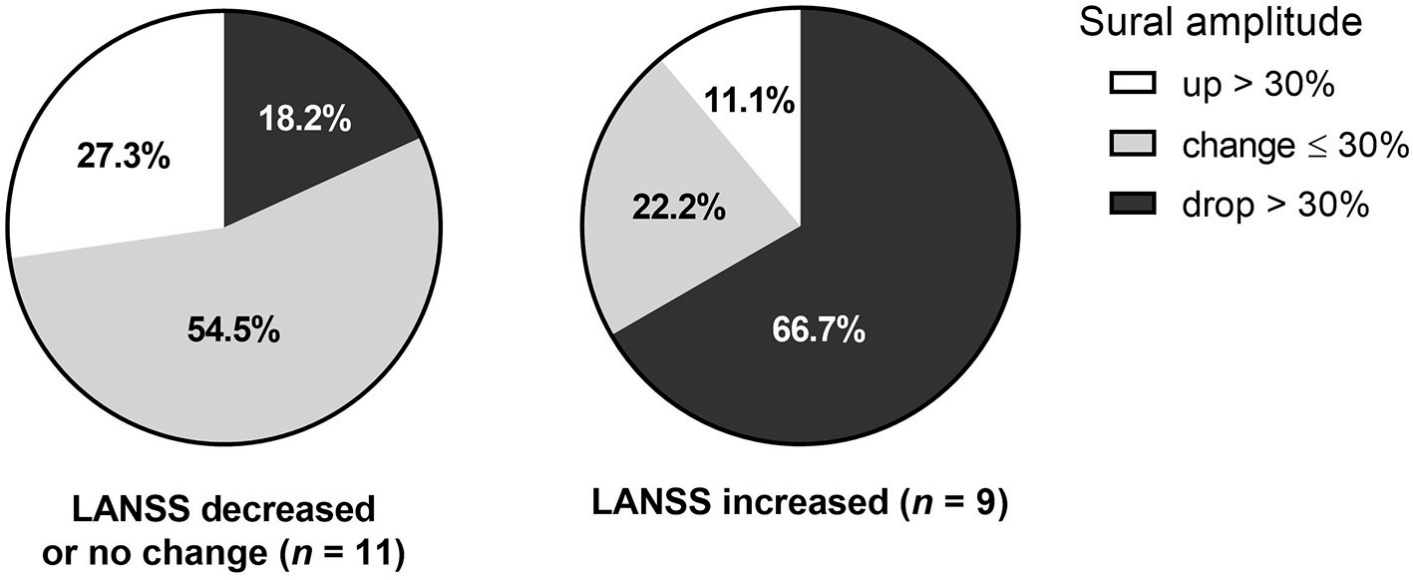
Change of sural amplitude in relation to symptomatic change.The change of sural amplitude was associated with change of LANSS score. The LANSS score decrease, or no change was associated with follow-up sural amplitude change of less than or equal to 30% or over 30% increase. On the other hand, LANSS score increase or worsening of symptoms were most frequently observed in those with sural amplitude drop over 30%.

Among 20 patients who underwent FU studies, the initial NCS results were indicative of CIPN in only six patients (30%). In the FU NCS, additional five subjects showed positive NCS results comparable to CIPN that the sensitivity of the NCS for CIPN increased to 55%. On the other hand, all 20 patients with FU NCS were compatible with CIPN by LANSS initially (16.0 ± 2.0) and at FU (16.3 ± 2.0); however, there was no significant difference between the initial and FU LANSS scores (Table 1). In comparison between the initial and FU sural SNAP amplitude, 8 out of 20 (40.0%) subjects showed more than a 30% drop of sural amplitude (Criterion B). The sensitivity of NCS increased to 65% when including either sural amplitude < 10 μV (<3 μV for 60 s, <1 μV for 70 s) or >30% drop of amplitude in FU studies (Table 2). Six of eight patients (75%) with > 30% drop of amplitude showed aggravation of LANSS score. On the other hand, 75% of those with >30% increase or within 30% change in sural amplitude had improved or no aggravation of symptoms (Figure 1).

**Table 2:** Change of NCS Sensitivity for CIPN diagnosis according to sural amplitude difference (*N* = 20).

**Diagnostic Criteria**	***N***	**Sensitivity (%)**
A. Sural amplitude <10 μV (60 s <3 μV, 70 s <1 μV)		
Initial	6	30.0
FU	11	55.0
B. Sural amplitude > 30% drop at FU	8	40.0
C. Either A or B criteria at FU	13	65.0

*NCS, nerve conduction study; CIPN, chemotherapy induced peripheral neuropathy; FU, Follow-up*.

## Discussions

In this study, cross-sectional NCS results of 88 patients showed low sensitivity (69.7%) and specificity (47.2%) for the diagnosis of CIPN. However, the FU NCS performed in some patients revealed progressive electrophysiological deterioration (Criterion A or B), without significant symptom aggravation by LANSS. This ongoing nerve demise prior to symptom exacerbation suggests that serial NCS follow-ups can be helpful in earlier detection of electrophysiological changes, which will further increase the chance to mitigate or treat additional neural impairment and symptom aggravation.

Although CIPN is one of the notorious dose-limiting factors for cancer therapy, early diagnosis of CIPN is considered under-reported and under-recognized possibly due to often reported indifference between subjective CIPN symptoms and standardized diagnostic methods (18), limiting quantifiable medical decision making. The standard neurophysiological test used for diagnosis of CIPN is NCS including sensory and motor conduction velocity, sensory nerve action potential, and compound muscle action potential, and needle electromyography (19), which can exclude other neuropathic etiologies (20).

NCS is preserved in younger age and electrophysiological abnormalities in relation to clinical symptoms are less likely to be detected in people under the age of 50. The effect of aging in the decrease of SNAP amplitude has been established (21), and the age-group difference was counted in the analysis of this study by using different reference for each age group. Regardless of age, the sensitivity of initial NCS in CIPN diagnosis was low, however, showed the tendency to increase in the FU study (Table 2). Therefore, the interpretation of absolute NCS results should be followed by serial NCS if symptoms persist.

Early decline of SNAP prior to symptom development or functional impairment has been suggested as indicator of subsequent CIPN development (14, 22). However, CIPN suspected patients are mostly referred after the onset of symptoms and the NCS are conducted thereafter. When NCS is performed once in such patients, low sensitivity is evident as in this study: among 88 clinical suspected CIPN patients, results of only 26 patients were strictly compatible to abnormal sural amplitude. The low sensitivity of NCS may lead to cases where the NCS result is normal even when possible peripheral neuropathy is suspected after chemotherapy in actual clinical practice (23). Even if neuronal damage progresses, a single NCS result is likely to remain within the reference values due to wide range of normal references. Therefore, the application of standard NCS diagnostic criteria may have limited utility in such patients. Patients with risk factors for CIPN including prior therapy with a neurotoxic agent, diabetes mellitus, folate/vitamin B12 deficiencies, African race, and older age (7, 24) needs to be informed about CIPN and monitored carefully with baseline NCS and serial FU studies. This will not only enhance the sensitivity of NCS test but also provide prognostic outcome as well as evidence of early treatment.

In addition, clinical diagnosis based on a single test at one-time point can be inconclusive since chemotherapy may last for months with possible continuous and cumulative nerve damage. In a study of serial sensory NCS FU after initiation of paclitaxel, a taxane, significant reduction of SNAP amplitude continued over the course of 8-week treatment (14). Also, persistent reduction of sensory amplitudes was associated with severer symptoms assessed by Neuropathy Symptom Scale, indicating worse outcome of CIPN (Figure 1). It can be inferred that in patients with suspected CIPN, abnormal symptomatic progression may eventually follow even if the results of a single test at one-time point are within normal range. In other words, longitudinal serial tests may increase the diagnostic accuracy and provide further clues for treatment direction and outcome with apparent degree of nerve injury.

The FU NCS results of sural amplitude decrease suggests continuous progression of neural injury similar to a previous study in which sural amplitude decrease was consistently observed with continuous nerve injury even after the termination of chemotherapy (14). Moreover, reduction of SNAP amplitude is associated with continuous symptom progression following cessation of neurotoxic chemotherapy (25).

Change of CIPN symptoms reflected on LANSS score was statistically insignificant despite of significant sural amplitude change. The sensory tests in LANSS score consist of pinprick and light touch without vibration sense. In a previous study evaluating the relationship between clinical measures and NCS in CIPN, vibration of lower limbs showed the most significant association with abnormal sural nerve amplitudes, whereas pin prick did not show equivalent strength for clinical use (9). Although the LANSS score assesses both the large, myelinated nerve fibers (Aβ for light touch) and small, thinly myelinated fibers (Aδ for pin prick), overall sensory dysfunction depicted may not have been as sensitive as that using vibration sense. Moreover, detecting sensory dysfunction relative to the contralateral side or non-painful area could have been less sensitive as the minimum meaningful differences may differ individually (26). Therefore, assessing CIPN only based on clinical features has limitations that finding a practical consensus relating neurophysiological test and clinical assessments is needed.

Clinical symptoms tended to change relative to the degree of sural amplitude changes. Among those with sural amplitude drop > 30%, six out of eight patients (75%) showed LANSS score increase indicating symptomatic aggravation. Meanwhile among those with over 30% increase of amplitude, LANSS score remained without change in 75% (*N* = 3). Among the others with <30% change of sural amplitude, six of eight patients (75%) had no change or improved LANSS score (Figure 1). This finding may suggest that greater sural amplitude increase is likely to denote symptomatic improvements while minimal amplitude change indicates no further symptomatic aggravation. However, further study with more patients is needed to confirm the correlation of electrophysiological change and clinical prognosis.

There are several limitations in this study. One is the small number of FU NCS and that it was mostly conducted for those with severe clinical symptoms. However, the selective FU NCS in this study also reflects current NCS usage in the clinical practice of cancer patients in which minor electrophysiological changes can remain unnoticed. Further study with large sample size will be helpful to increase the sensitivity of NCS in CIPN-prone patients. Secondly, the association of chemotherapy dose and duration with CIPN symptoms was not assessed in this study. Moreover, the use of concomitant CIPN-prone agents including platin regimen was not excluded that the synergistic effect of those agents on serial NCS cannot be discarded and that chemotherapy regimen-specific effect needs to be addressed in future studies. Lastly, NCS can be normal during early stages of the axonopathy since it monitors the amplitude and velocity of large, myelinated fibers. As CIPN involves multiple areas of peripheral nervous system and any peripheral nerve fiber types including Aβ, Aδ, and unmyelinated C fibers (25, 27), additive quantitative sensory testing to assess Aδ and C fiber may provide further information. Moreover, comparison study of NCS and other clinical assessments may provide standard criteria for diagnostic and therapeutic monitoring of CIPN.

In conclusion, a single NCS at one point has a limited value in clinically suspected CIPN. Serial NCS during chemotherapy may be helpful in attaining the objective evidence of CIPN and assessing the progression of chemotherapy induced nerve damage and to monitor the course of CIPN especially in young age. In CIPN-prone patients with electrophysiological evidence of deterioration, future studies utilizing neuroprotective medications and rehabilitation with serial NCS FU may provide further evidence of serial NCS monitoring for early treatment.

## Data Availability Statement

The raw data supporting the conclusions of this article will be made available by the authors, without undue reservation.

## Ethics Statement

Given the retrospective study design, the informed consent from patients in this research was waived by the institutional review board of Yonsei University Health System (ethical approval number: 4-2018-0990) and the study protocol was reviewed and approved by the Institutional Review Board of Yonsei University Health System (ethical approval number: 4-2018-0990). Written informed consent for participation was not required for this study in accordance with the national legislation and the institutional requirements.

## Author Contributions

KHC drafted and completed the manuscript. EYH substantially contributed to data analysis and interpretation. JCS participated in statistical analysis and writing. MCH, KHA, and SHC collected data and participated in clinical assessments. SHI designed the study and is responsible for the integrity and accuracy of the data and writing. All authors contributed to the article and approved the submitted version.

## Funding

This study is supported by a research grant of Research institute of Rehabilitation Medicine Yonsei University College of Medicine for 2020.

The work was supported by the National Research Foundation of Korea (NRF) grant funded by the Korea government (MSIT; Ministry of Science & ICT) (No. 2020R1G1A110314811).

## Conflict of Interest

The authors declare that the research was conducted in the absence of any commercial or financial relationships that could be construed as a potential conflict ofinterest.

## Publisher's Note

All claims expressed in this article are solely those of the authors and do not necessarily represent those of their affiliated organizations, or those of the publisher, the editors and the reviewers. Any product that may be evaluated in this article, or claim that may be made by its manufacturer, is not guaranteed or endorsed by the publisher.
